# Cell-type-specific Jumonji histone demethylase gene expression in the healthy rat CNS: detection by a novel flow cytometry method

**DOI:** 10.1042/AN20130050

**Published:** 2014-05-27

**Authors:** Stephanie M.C. Smith, Rebecca S. Kimyon, Jyoti J. Watters

**Affiliations:** *Department of Comparative Biosciences, University of Wisconsin, 2015 Linden Dr., Madison, WI 53706, U.S.A.

**Keywords:** adult, cell sorting, epigenetic, gene expression, miRNA, zinc fixation, CNS, central nervous system, DAPI, 4′,6-diamidino-2-phenylindole, DEPC, diethyl pyrocarbonate (q.v.), EAAT2, excitatory amino-acid transporter 2, FCM, flow cytometry, FSC, forward scatter, GFAP, glial fibrillary acidic protein, GLT-1, glutamate transporter 1, H3K27, H3 lysine 27, H3K27me1, monomethylated H3K27, H3K27me3, trimethylated H3K27, HBSS, Hanks balanced salt solution, JmjC, Jumonji C, miRNAs, microRNAs, mZBF, modified zinc-based fixative, NeuN, neuronal nuclei, PE, R-phycoerythrin, PE-Cy7, R-phycoerythrin-cyanine 7, SSC, side scatter, ZBF, zinc-based fixative

## Abstract

Our understanding of how histone demethylation contributes to the regulation of basal gene expression in the brain is largely unknown in any injury model, and especially in the healthy adult brain. Although Jumonji genes are often regulated transcriptionally, cell-specific gene expression of Jumonji histone demethylases in the brain remains poorly understood. Thus, in the present study we profiled the mRNA levels of 26 Jumonji genes in microglia (CD11b^+^), neurons (NeuN^+^) and astrocytes (GFAP^+^) from the healthy adult rat brain. We optimized a method combining a mZBF (modified zinc-based fixative) and FCM (flow cytometry) to simultaneously sort cells from non-transgenic animals. We evaluated cell-surface, intracellular and nuclear proteins, including histones, as well as messenger- and micro-RNAs in different cell types simultaneously from a single-sorted sample. We found that 12 Jumonji genes were differentially expressed between adult microglia, neurons and astrocytes. While *JMJD2D* was neuron-restricted, *PHF8* and *JMJD1C* were expressed in all three cell types although the expression was highest in neurons. *JMJD3* and *JMJD5* were expressed in all cell types, but were highly enriched in microglia; astrocytes had the lowest expression of *UTX* and *JHDM1D*. Levels of global H3K27 (H3 lysine 27) methylation varied among cell types and appeared to be lowest in microglia, indicating that differences in basal gene expression of specific Jumonji histone demethylases may contribute to cell-specific gene expression in the CNS (central nervous system). This multiparametric technique will be valuable for simultaneously assaying chromatin modifications and gene regulation in the adult CNS.

## Abstract 2

Neurons, microglia and astrocytes were sorted from the non-transgenic adult brain. Cell-specific microRNAs, Jumonji histone demethylase gene expression and histone modifications were assessed. This method will enable new understanding of CNS regulation of gene expression and epigenetic modifiers *in vivo*.

## INTRODUCTION

The CNS (central nervous system) is comprised highly interconnected, yet functionally heterogeneous CNS cell populations. Understanding how these cells function both individually and within a network is crucial to unraveling their role in health and disease. Interpretation of many existing epigenetic and gene regulation studies in the adult CNS are complicated by the fact that they are typically performed on tissue homogenates, so the effects within individual cell types cannot be distinguished. Another major technical hurdle in evaluating gene expression *in vivo* is the inability to efficiently isolate nucleic acids (RNA) from paraformaldehyde-fixed and sorted cells since major CNS cell types are best characterized by proteins expressed intracellularly. As a result, investigations of gene regulation in the brain often utilize cultured CNS cells that are usually derived from late embryonic or neonatal animals, confounding understanding of these processes in the adult. Laser capture microdissection (Vincent et al., 2002; Luo et al., 2007), live cell sorting by FCM (flow cytometry) from transgenic animals expressing fluorescent proteins driven by cell-type-specific promoters (Lobo et al., 2006), ribosomal-tagging for mRNA isolation from transgenic animals (Doyle et al., 2008; Heiman et al., 2008; Sanz et al., 2009), and alcohol-based fixation to sort neurons for subsequent RNA analysis (Guez-Barber et al., 2011a) are commonly used methods permitting assessments of RNA in specific CNS cell populations, but each have their own drawbacks. Although each technique has enabled significant advances in neurobiology, their limitations include investigations of only a single-cell type at a time, the need to use and maintain transgenic animals, and/or the inability to concurrently analyze nucleic acids and intracellular proteins in a single sample. Therefore, we endeavored to overcome these obstacles by optimizing a novel method using non-transgenic, adult rats where proteins and nucleic acids can be concurrently analyzed by FCM in multiple neuron and glial cell types simultaneously identified using a combination of intracellular and extracellular markers.

Although FCM is commonly used to analyze and sort pure cell populations, the ability to efficiently recover nucleic acids from formaldehyde-fixed cells is not (Diez et al., 1999). This limitation is particularly significant for neuroscience research because the best-characterized cell-type-specific markers for neurons and astrocytes are intracellular, thus requiring fixation and permeabilization for immunostaining-based detection. Guez-Barber and colleagues (Guez-Barber et al., 2011a) reported the use of an alcohol-based fixative to sort neurons from non-transgenic animals for subsequent RNA analysis, the utility of which has been demonstrated several times for evaluating nucleic acids in sorted neurons (Guez-Barber et al., 2012; Fanous et al., 2013; Liu et al., 2014). However, when endeavoring to isolate nucleic acids from identified and sorted neuron and glial cell populations simultaneously, based on a combination of intracellular and cell surface identification markers, alcohol fixation was ineffective in our studies. We thus turned to a ZBF (zinc-based fixative) which was previously shown to preserve cellular structure, proteins and nucleic acids in histological and cellular studies (Wester et al., 2003; Lykidis et al., 2007; Jensen et al., 2010). Because a mZBF (modified zinc-based fixative) was previously shown to preserve nucleic acids better than the standard zinc fixation methods (Lykidis et al., 2007), we evaluated intracellular, extracellular and nuclear proteins, as well as post-translational modifications to histone tails with mZBF after a mechanical dissociation protocol. We found that all parameters were readily preserved. Fixed microglia, neurons and astrocytes were sorted based on the cell surface (CD11b) and intracellular markers [NeuN (neuronal nuclei) and GFAP (glial fibrillary acidic protein), respectively], and we obtained high-quality messenger and small non-coding RNAs [miRNAs (microRNAs)]. We also observed differences in basal histone H3K27 (H3 lysine 27) methylation status among cell types, suggesting fundamental differences in chromatin structure between CNS cell types. The purity of sorted cell populations from the adult CNS was confirmed by evaluation of mRNA levels of cell-type-specific genes in individual cell populations.

The importance of histone demethylation in the molecular regulation of CNS gene transcription is becoming increasingly appreciated. Our overall research goal is to understand the role of histone demethylation in regulating gene transcription in individual CNS cell types (microglia, neurons and astrocytes), since cell-specific gene regulation strongly contributes to cell–cell communication in CNS health and disease. Two families of histone demethylases have been identified: LSD (lysine-specific demethylases) and JmjC (Jumonji C) domain family proteins (Kooistra and Helin, 2012). Whereas the Jumonji demethylases comprise the largest gene family of histone demethylases, little is known about their expression or function in the adult CNS, in any cell type. To begin to understand how epigenetic regulation influences CNS cell function, and because so little is known about the expression of key Jumonji demethylases in any CNS cell type in the adult, we used the present method to independently profile the expression of the 26 best characterized Jumonji histone demethylase family members in microglia, neurons and astrocytes. We found that seven Jumonji genes had greater than a 3-fold change in mRNA expression levels between cell types. Of these, *PHF8*, *JMJD1C* and *JMJD2D* had neuron-specific expression, whereas *JMJD3* and *JMJD5* were primarily expressed in microglia. *UTX* and *JHDM1D* had very low expression in astrocytes compared to neurons and microglia. Collectively, these data suggest that there is cell-type-specific regulation of basal histone demethylase expression in the CNS. Moreover, because these enzymes have different histone targets and enzymatic specificities (Kooistra and Helin, 2012), these results also suggest that histone demethylation likely plays different functional roles in the control of gene expression in neurons, astrocytes and microglia in the healthy brain. The present technique provides a simple experimental means with which to begin *in vivo* studies of cell-specific epigenetic gene regulation in multiple CNS cell types simultaneously.

## MATERIALS AND METHODS

### Animals

This study was carried out in strict accordance with the recommendations in the Guide for the Care and Use of Laboratory Animals of the National Institutes of Health. Experiments were performed on adult, male, Sprague–Dawley rats (Harlan, Indianapolis, IN) weighing 300±50 g. Rats were housed and handled in accordance with the Guide for Care and Use of Laboratory Animals in an AAALAC-accredited facility. All surgical and experimental procedures were approved by the University of Wisconsin, Madison Institutional Animal Care and Use Committee. All efforts were made to minimize the number of animals used and their suffering.

### Materials

HBSS (Hanks balanced salt solution) was purchased from Cellgro. Calcium acetate, zinc chloride, zinc trifluroacetate, glycerol, DEPC [diethyl pyrocarbonate (q.v.)], EDTA and TRI Reagent were purchased from Sigma Aldrich. Percoll was purchased from GE Healthcare. DNase was purchased from Worthington Biochemicals. Lightning Link Antibody conjugation kit was purchased from Novus. Glycoblue reagent was purchased from Ambion. NCode™ VILO™ miRNA cDNA Synthesis kit, RNase AWAY and DAPI (4′,6-diamidino-2-phenylindole) were purchased from Invitrogen. Primers were designed using Primer 3 software and were purchased from Integrated DNA Technologies. Power SYBR green was purchased from Applied Biosystems. CD11b and GFAP antibodies were purchased from BD Biosciences. NeuN, EAAT2 (excitatory amino-acid transporter 2) [GLT-1 (glutamate transporter 1)], H3K27me3 (trimethylated H3K27), H3 and the rabbit IgG isotype control antibodies were purchased from Millipore. The H3K27me1 (monomethylated H3K27) antibody was purchased from Epigentek. The goat anti-rabbit PE-CY7 (R-phycoerythrin-cyanine 7) antibody was purchased from Santa Cruz Biotechnology.

### Control of RNAse and DNAse contamination

Special precautions were taken during sample collection and processing to preserve RNA integrity. Only certified nuclease-free plastic ware and/or glassware baked at 400°C for 4 h were used. All surfaces and tools were treated with RNase AWAY (Invitrogen) to prevent RNase contamination. All solutions were prepared with DEPC-treated water.

### Mechanical dissociation and fixation of neural tissue

Rats were killed with an overdose of isoflurane anesthetic, and perfused with cold 0.1 M PBS. The brain, excluding olfactory bulbs, brainstem and cerebellum was dissected, and placed into cold HBSS on ice. Samples remained on ice for the duration of the procedure, and all centrifugation steps were performed at 4°C. We modified a mechanical dissociation protocol (Chassevent et al., 1984) for creating single-cell suspensions of CNS tissues by pushing the tissue through a pre-moistened 100 μm filter with a syringe plunger and flushing the filter with ice-cold HBSS supplemented with 0.01 mg/ml DNase and 0.1 mM EDTA. Myelin was removed by high-speed centrifugation at 850 ***g*** for 15 min in a solution consisting of 26% Percoll in 0.1 M PBS. Dissociated cells were washed in ice-cold HBSS and pelleted by centrifugation at 350 ***g*** for 5 min. Cells were resuspened in a mZBF (0.5% zinc chloride, 0.5% zinc trifluroacetate, 0.05% calcium acetate in 0.1 M Tris–HCL, pH 6.4–6.7) and glycerol (1:1), as previously described (Lykidis et al., 2007; Jensen et al., 2010). Samples were stored at −20°C overnight or until ready to be used. Consistent with previous reports (Jensen et al., 2010), we have stored samples for several weeks with no detectable loss of cell integrity or immunostaining efficiency (results not shown). To assess the effects of the fixation process on RNA quality, comparisons between fresh and fixed tissues, and fixed/sorted cells were performed.

### Cell population analysis by FCM

Fixed samples were washed 3X in ice-cold PBS and permeabilized on ice for 20 min in 1X PBS+0.2% saponin+0.1% (w/v) BSA. All staining occurred on ice in the permeabilization buffer, protected from light. Cells were stained with anti-NeuN-Alexa 488 (1:200), anti-CD11b-PE-Cy7 or PE (R-phycoerythrin) (1:200) and anti-GFAP-Alexa 647 (1:50) or anti-EAAT2 (GLT-1)-Alexa 647 (1:100) antibodies for 45 min on ice. Prior to staining, the EAAT2 (GLT-1) antibody was custom conjugated to Alexa 647 utilizing the Lightning-Link antibody conjugation kit, according to the manufacturer's instructions. Samples were washed and resuspended for sorting in permeabilization buffer containing DAPI (1 μg/ml) to identify cells with intact nuclei. FCM analysis and cell sorting was performed using a FACSAria II equipped with 350-, 405-, 433-, 532- and 633-nm lasers, a standard filter set, and FACSDiva software (BD Biosciences). All appropriate compensation and FMO controls were performed and utilized in the analysis. Intact cells were identified using FSC (forward-scatter) and SSC (side-scatter) parameters, singlet gates, and DAPI staining to identify cells in cell cycle at the time of fixation. Samples were first gated to exclude doublets and any events off scale with the following singlet gates: FSC-Width/SSC-Area, SSC-Width/FSC-Area. We then used a cell cycle gate to identify cells with intact nuclei based on DAPI-Width/DAPI-Area plotted on a linear scale. Cell populations were identified and gated using FMO controls. Cells were identified based on their fluorescence properties, and were sorted through a 120 μM nozzle at 14 psi into 1.5 ml Eppendorf tubes. Sorted cell populations were defined as the following: neurons (NeuN^+^/CD11b^−^/GFAP or GLT-1^−^), microglia (CD11b^+^/NeuN^−^/GFAP or GLT-1^−^) and astrocytes (GFAP or GLT-1^+^/CD11b^−^/NeuN^−^). Immediately following the sort, the cells were pelleted and resuspended in Tri Reagent for RNA isolation. Following the sort, an aliquot of the remaining, unsorted, fixed and stained cells was taken for comparison to the unfixed and fixed/sorted cells. Post-sort population analyses and graphical representations were performed in FlowJo software v.10. (TreeStar Inc.). Data are representative of three separate experiments with four independent samples per experiment.

### Cell-type identification on ImageStream

Cortical tissue was mechanically dissociated into a single-cell suspension, fixed in mZBF, and stained with CD11b-PE, NeuN-Alexa488, GFAP-Alexa647 and DAPI as described above. Fluorescent images were visualized on the Amnis ImageStream. Cellular expression and distribution of these cell identifiers were analyzed in ≥20 000 cells at 40× magnification, using IDEAS software (Amnis).

### Detection of histone-tail post-translational modifications

To test whether the mZBF is compatible with detection of histone-tail post-translational modifications by flow cytomery, samples were prepared as described in the previous sections, with the following modifications. Cells were stained with anti-NeuN-Alexa 488 (1:200), anti-GFAP-Alexa 647 (1:50), anti-CD11b-PE (1:200), and rabbit-anti-H3K27me3 (1:100), rabbit-anti-H3K27me1 (1:100), or rabbit-anti-H3 (1:100) antibodies for 45 min on ice. Samples were washed, and stained on ice for 30 min with an anti-rabbit-PE-Cy7 (1:750) secondary antibody. Following incubation with the secondary antibody, the cells were washed and resuspended in permeabilization buffer containing DAPI (1 μg/ml). The cells were analyzed on a BD LSR II equipped with 350, 405, 433, 561 and 642 nm lasers, a standard filter set, and FACSDiva software (BD Biosciences). All single stain compensations and FMO controls were performed and utilized in the analysis. In single stains and FMO control samples, we included rabbit IgG isotype control (1:100) for the histone antibodies plus secondary antibodies when appropriate, to control for non-specific antibody binding. A total of 250 000 events were collected for each sample. Populations were identified and defined as previously described, and the median fluorescent intensity for H3, H3K27me3 and H3K27me1 were calculated for each cell population. All data analyses were performed using FlowJo software v. 10 (TreeStar Inc.). Data are representative of four independent rat brain samples.

### DNA, mRNA and miRNA extraction/reverse transcription

Total RNA was extracted from fresh, fixed and fixed/sorted cells according to the Tri Reagent protocol, with the addition of Glycoblue during the isopropanol incubation for RNA isolation. cDNA was synthesized from 0.25 μg of total RNA using the NCode™ VILO™ miRNA cDNA Synthesis kit according to the manufacturer's protocol. This enables amplification of both mRNA and miRNAs by qPCR.

### Custom Jumonji histone demethylase arrays

A custom Jumonji qRT-PCR array was performed in duplicate on pooled samples (*n*=4/pool) for the initial broad screen. mRNA expression levels of 26 characterized Jumonji genes were present on the array and results were normalized to the average C_T_ of the control genes 18s and β-tubulin, using the delta C_T_ method (Livak and Schmittgen, 2001). The expression of a given gene was averaged across all cell types, and the expression within a given cell type was normalized to this average. The normalized values were subjected to a Log_2_ transformation and were graphically displayed as a heat map created with TreeView software (Saldanha, 2004). All genes whose expression differed among cell types by greater than 3-fold were independently confirmed by gene-specific qRT-PCR in each of four independent sorted samples, derived from four different animals.

### qRT-PCR (quantitative real-time PCR)

qRT-PCR was performed on cDNA using Power SYBR Green and quantified using an ABI 7500 Fast system. Primers were designed using Primer 3 software and specificity of target gene amplification was confirmed by NCBI BLAST searches and verification that the dissociation curves had a single peak with a T_m_ consistent with the expected amplicon. Primer efficiency was tested through the use of serial dilutions. Genes were considered non-detectible if the C_T_ value fell outside of the linear range of the given primer set serial dilution curve. Values from duplicate qPCR measurements were averaged, normalized to the average of two control genes (18s and β-tubulin, for mRNA analyses; and snoRNA135 and snoRNA234, for miRNA analyses), and relative expression was determined using the delta C_T_ method. Primer sequences are provided in Table 1.

**Table 1:** Primer table The table includes the primer sequences used to assess gene expression by qPCR, the associated protein name, gene name and the NCBI accession number for the gene on which the primer sequences are based.

Protein name	Gene name	NCBI reference sequence	Forward primer (5′)	Reverse primer (5′)
	18s	M11188.1	CGG GTG CTC TTA GCT GAG TGT CCC G	CTC GGG CCT GCT TTG AAC AC
β-Tubulin	Tubb2a	NM_001109119.1	AGA CCA TGC TGG AGG ACA ACA	AGG ATG CCA CGG CTG ATG
NeuN	Rbfox3	NM_001134498.2	TGC CAA TGG CTG GAA GCT AA	TAG GGG AAA CTG GTC ACT GC
Neurofilament-heavy chain	Nefh	NM_012607.2	GGC CTC CTA CCA GGA TGC AAT TCA G	TGC GCG GCC ATC TCC CAT TT
β-III Tubulin	Tubb3	NM_139254.2	TAG CCG AGT GAA GTC AGC ATG AGG G	ACC TCC CAG AAC TTG GCC CCT ATC
cd11b	Itgam	NM_012711.1	GGG CAG GAG ACG TTT GTG AA	TGC CCA CAA TGA GTG GTA CAG
Iba-1	Aif1	NM_017196.3	TCA TCG TCA TCT CCC CAC CT	GCT TTT CCT CCC TGC AAA TCC
CD68	CD68	NM_001031638.1	GGA CTA ATG GTT CCC AGC CA	TGG GTC AGG TAC AAG ATG CG
GFAP	GFAP	NM_017009.2	GGG CGA AGA AAA CCG CAT CAC CA	ATG ACC TCG CCA TCC CGC ATC
ALDH1L1	Aldh1l1	NM_022547.1	CCT GGA CAC TGG TGA CCT TC	ACG ATT GTA CAG CGT GCT CA
GLT-1	Slc1a2	NM_017215.2	GCA TCA ACC GAG GGT GCC AAC AA	CCC AGG TTT CGG TGC TTT GGC T
C2orf60	Tyw5	NM_001170473.1	TTA CCC GCA GAG AAA GCC TC	ACT GCA GCG ACG TGA ATC TT
Jarid1A	Kdm5a	NM_001277177.1	TGA ACT GTC TTC TGC CCT GG	AGT GTC CCT GTA AGT CTG GAT TG
Jarid1B	Kdm5b	NM_001107177.1	GCC ACC ATT CGC TTG TGA TG	TTA CAC GTG TTT GGG CCT CC
Jarid1C	Kdm5c	XM_241817.7	GGT TCC TTG CTA CGC TCT CA	TAC ACT GCA CAA GGT TGG CT
Jarid1D	Kdm5d	FJ775729.1	ATG CCT CTG CAA CCT CCA TC	AGA TCA CAC CGC AGA GCT TC
Jarid2	Jarid2	XM_003752957.1	GCA GGC GAA TCT GGT TTT GG	GCT GAT TGC AAA AGG GGA CA
JHDM1A	Kdm2a	NM_001108515.1	GCA TCC CTG GAG TGG TTT CT	TAC CAC GCA ATC TCT GGC TG
JHDM1B	Kdm2b	NM_001100679.1	CTT TCC CCC TCC GCC AAA AT	GTC GTA TCT CTG GCG GTC AAT
JHDM1D	Jhdm1d	XM_003749720.1	TGA TGG CTC CAA ACC TGT TCA	TTC ATC GGC ACT TGG GAA GAC
JMJD1A	Kdm3a	NM_175764.2	TTG CTC TGA GGT CTC TCC CA	GCA GTA CAG CCA AGC AGG AT
JMJD1B	Kdm3b	XM_001061636.2	GGA CCT AGC GAT CTT TGT GGA	AGC GTG AAC CTT AAC CCA GG
JMJD1C	Jmjd1c	NM_001191719.1	TGC GCT GAC CTT CAA ACC AT	GTT CGG GCT TTA GGC TGT CT
JMJD2A	Kdm4a	NM_001107966.1	AAA GAC AGT GGG ATC GGC G	ACC TGG AGC CTA AAG CCC TA
JMJD2B	Kdm4b	NM_001044236.2	ACT GCG CTG GAT CGA CTA TG	GCT GCA GGA TGC GTA CAA AC
JMJD2C	Kdm4c	NM_001106663.2	TGG AGA GTC CCC TAA ATC CCA	TTG GCA AGA CCT GCT CGA TG
JMJD2D	Kdm4d	NM_001079712.1	AGG CGC AAA TAA GTA CGG GG	GGG GTG CAG CAG ATT CTC TT
JMJD3	Kdm6B	NM_001108829.1	CAA ACC CCC GCT TTT CTG TG’	ATT TGG GTG GCA GGA GGA GG
JMJD4	Jmjd4	NM_001105784.1	AGG GAG GCT ACT CCT CTC CAA	ATC CAC CAA GGA GTC TCT GC
JMJD5	Kdm8	NM_001037196.1	CCG TGG AAG TGG GTT CAA GA	CAT CCT TTG CCT CGC TCA GA
JMJD6	Jmjd6	NM_001012143.2	TAG CAG CTA TGG CGA ACA CC	CCC CAT CAC AAA CCA CCT GTA
JMJD7	Jmjd7	NM_001114656.1	TGC TCG CGA CCT CAA TGT A	GGT AGA AGC AGA GCG GAC TT
JMJD8	Jmjd8	NM_001014116.1	TGG ACG ATT CGG TCT GCT TT	ACT CTG TTT CCA TCC CCC TTC
Mina53	Mina53	NM_153309.2	ATG CCA AAG AAA GTG AAG CCC	GTA GCT CCT CTT TCA CCT GCT
PHF2	Phf2	NM_001107342.1	TCA GAC ACC AGA ATG TCC AGC	TCG GGC CAG TAG TTT TCC AC
PHF8	Phf8	NM_001108253.1	TTT GGG ACC GTG GAC GTT T	GTC AGA AAG GCA GCA ACA AGC
UTX	Kdm6a	NM_009483.1	CCA CCC TGC CTA GCA ATT CA	CCA CCT GAG GTA GCA GTG TG
UTX	Uty	NM_009484	ATT ATC TCT CAC TAC TGC TGC CC	CGA AGA AGC TGC TGT CTA ATC CAC
	snoRNA135/Snord65	NR_028541.1	AGT ACT TTT TGA ACC CTT TTC CA	
	snoRNA234/Snord70	NR_028554.1	TTA ACA AAA ATT CGT CAC TAC CA	
	mir-26	NR_029742.1	GGT TCA AGT AAT CCA GGA TAG GCT	
	mir-146a	NR_031892.1	TGA GAA CTG AAT TCC ATG GGT T	

### Statistical analyses

Statistical analyses were performed on delta C_T_ values using Sigma Plot 11.0 software. A one-way ANOVA followed by the Tukey's *post hoc* test was utilized when comparing multiple groups. The Student's *t* test was utilized when comparing two groups. Statistical significance was set at the 95% confidence limit (*P*<0.05). A single symbol above a bar represents *P*<0.05; two symbols *P*<0.01; and three symbols *P*<0.001. Quantitative data are expressed as the mean ± 1 S.E.M.

## RESULTS

### Mechanical dissociation and mZBF fixation is suitable for the processing of brain tissue for FCM

**Figure 1:**
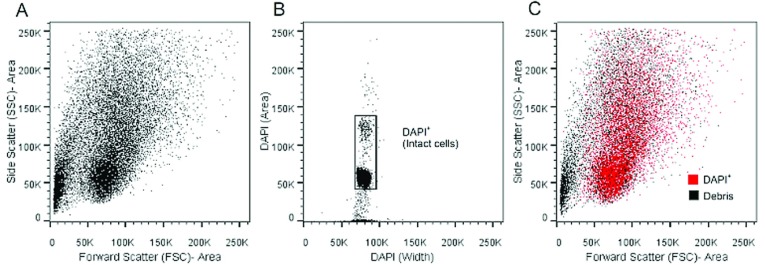
FSC/SSC and DAPI staining identifies intact CNS cells Rat brain tissue was mechanically dissociated into a single-cell suspension, fixed in mZBF, and processed for FCM, as described in the Methods section. Doublets and off scale events were gated out based on FSC-Area/SSC-Width and SSC-Area/FSC-Width. All plots started with the same singlet gate. (**A**) Typical FSC/SSC plot for rat brain cells. (**B**) To remove dead cells and debris from the analysis, we used DAPI staining to gate on cells that were in the cell cycle at time of fixation. (**C**) Backgating on DAPI^+^ cells identified where debris and intact cells appeared on the FSC/SSC plot. Data shown are representative of four independent experiments.

Mechanically dissociated and fixed adult rat brain tissue samples were analyzed by FCM. The gating strategy involved gating out doublets and off scale events based on the singlet gates obtained using FSC-width/SSC-area and SSC-width/FSC-area. The plots shown in Figure 1 are from a single-sample representative of four independent samples analyzed on the same day. Cells are readily distinguished from debris based on DAPI staining and FSC–SSC properties (Figures 1A and 1B). There is a clear separation in the FSC–SSC plot of mechanically dissociated brain tissue (Figure 1A), representing a visible division between cells and debris. These results are consistent with a report performed on zinc-fixed epithelial cells, showing that ZBF preserves FSC–SSC properties (Jensen et al., 2010). We used DAPI to distinguish between debris and cells with intact nuclei/cells in the cell cycle at the time of fixation (Figure 1B). DAPI fluorescence was plotted as DAPI-Area/DAPI-Width to both identify the DAPI^+^ cells while also serving as an additional singlet gate. The DAPI^+^ cell population accounts for 49.2±3.1% (*n*=4) of the total number of events. The DAPI stain is essential, as it acts as an additional means to identify intact cells and to exclude debris from the sample (Figure 1C). This reduces background autofluoresence and signal from non-specifically bound antibody.

### Microglia, astrocytes and neurons can be simultaneously identified in the non-transgenic, adult CNS

**Figure 2:**
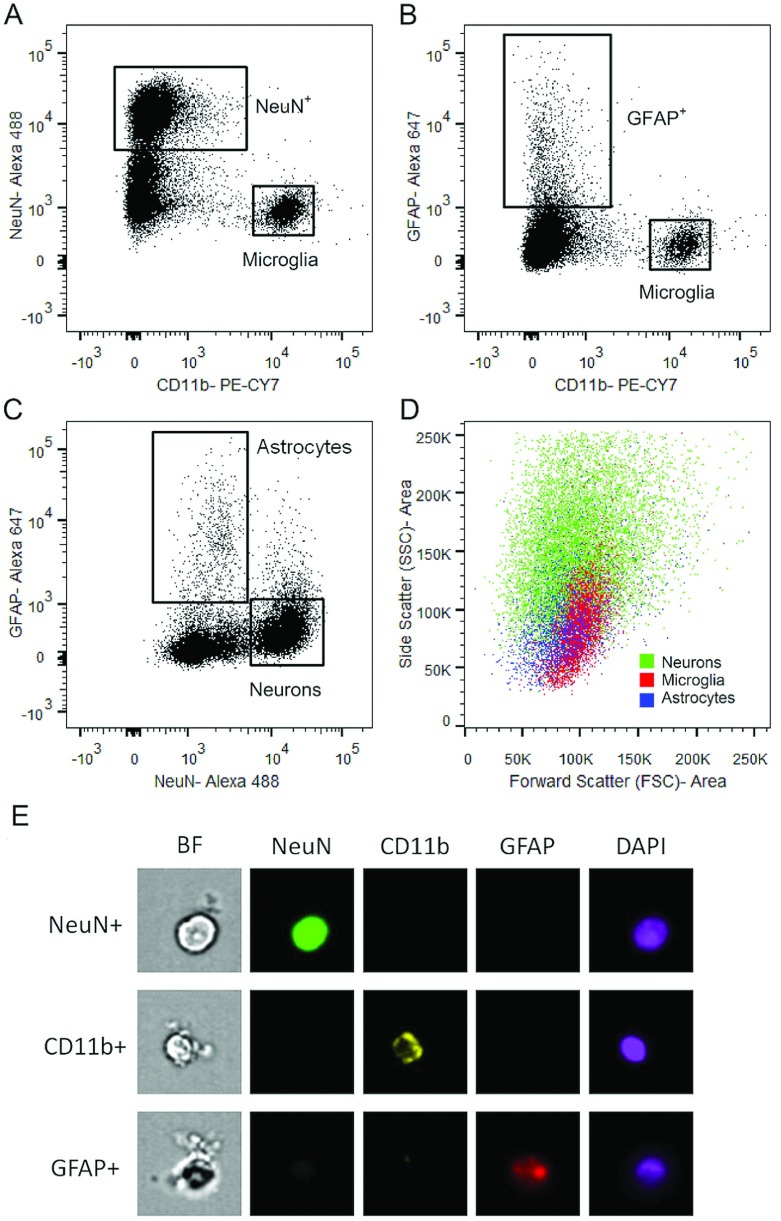
Neurons, microglia and astrocytes are simultaneously identified by FCM Single-cell mZBF-fixed suspensions were processed for FCM. Singlet cells were gated as described above, and then gated on DAPI as in Figure 1(B). DAPI^+^ neurons, microglia and astrocytes were identified using antibodies against the cell-specific markers NeuN (neurons; **A**,**C**), CD11b (microglia; **A**,**B**), and GFAP (astrocytes; **B**,**C**). (**C**) CD11b^−^ cells were plotted against GFAP and NeuN, revealing single positive GFAP and NeuN populations as well as a small GFAP and NeuN double positive population. (**D**) An overlay of the single positive CD11b, NeuN and GFAP populations reveals that they have overlapping and variable FSC/SSC properties indicating that these parameters alone are not useful on their own for cell-type identification in the CNS. The data shown are from a single sample that is representative of four independent experiments. (**E**) Representative images of DAPI^+^ cells staining single positive for CD11b, GFAP or NeuN from a single experiment of ≥20000 cells, at 40× magnification obtained using ImageStream analysis.

Dissociated and fixed samples were stained with antibodies to identify microglia (cell surface, anti-CD11b), astrocytes (intracellular, anti-GFAP or cell surface, anti-GLT-1) and neurons (intracellular, anti-NeuN). Distinct populations of DAPI^+^ microglia (Figure 2A), neurons (Figures 2A and 2C) and astrocytes (Figures 2B and 2C) were identified based on their immunofluorescent properties. There was clear separation between CD11b versus NeuN (Figure 2A) and CD11b versus GFAP (Figure 2B) immunostained cells. To determine if there was overlap between the NeuN and GFAP staining, we plotted NeuN versus GFAP in the CD11b^−^ cell population (Figure 2C). There were separate populations of cells that were single positive for NeuN (42.6%±2.29), GFAP (3.78%±0.28) and CD11b (11.3%±1.85) (*n*=4). However, there was also an unexpected population of cells that were double positive for NeuN and GFAP (0.94%±0.21, *n*=4). In addition, there was a population of DAPI^+^ cells that were triple negative for CD11b, NeuN and GFAP (39.0±1.3%, *n*=4). All CNS cells are not recognized by immunostaining with these three markers. On a per 100 mg of tissue basis, Table 2 summarizes the cell population frequencies, as well as the average number of cells of each type obtained in three separate and independent sample sorts. A wide FSC/SSC gate is necessary to encompass all neurons and astrocytes, and indicates that each cell type exhibits different but overlapping scatter properties (Figure 2D). Single positive CD11b-, GFAP- and NeuN-stained cells were visualized with an ImageStream analysis system where distribution and localization of immunostaining was evaluated in single cells (Figure 2E). Data shown in Figures 2(A)–2(D) are from a single-sample representative of four independent samples, analyzed on the same day.

**Table 2:** Expected cell numbers and cell-specific RNA quantification and purity from mZBF-fixed and sorted rat brain cells Half of a rat brain was weighed (~650 mg), mechanically dissociated, and processed for flow cytometry as described in the Methods. Doublets and off scale events were gated out based on FSC-Area/SSC-Width and SSC-Area/FSC-Width, and intact cells were gated based on DAPI positivity (Figure 1B). Single positive CD11b, NeuN and GFAP cells were sorted from the entire tissue sample. The total number of neurons (NeuN^+^), microglia (CD11b^+^) and astrocytes (GFAP^+^) obtained from the sort were normalized to tissue weight, and the total RNA isolated from the sorted cells was normalized to the number of cells sorted. The percent of DAPI^+^ cells, the number of cells obtained per 100 mg of tissue, the total RNA per 50 000 cells and the average RNA 260/280 ratios are presented in the table for each of the three-cell populations (average± the S.E.M, from *n*=4 independent samples).

	Percent of DAPI^+^ cells	Number of cells/100 mg tissue	RNA (ng)/50 000 cells	RNA 260/280
NeuN^+^	42.7±1.14	275.972±16.950	24.57±5.04	1.93±0.05
CD11b^+^	11.3±0.92	44.526±3.877	33.64±5.15	1.85±0.09
GFAP^+^	3.8±0.14	12.325±1.243	111.05±11.29	1.86±0.06

### mZBF permits retrieval of intact ribosomal-, messenger- and miRNAs from sorted CNS cells

**Figure 3:**
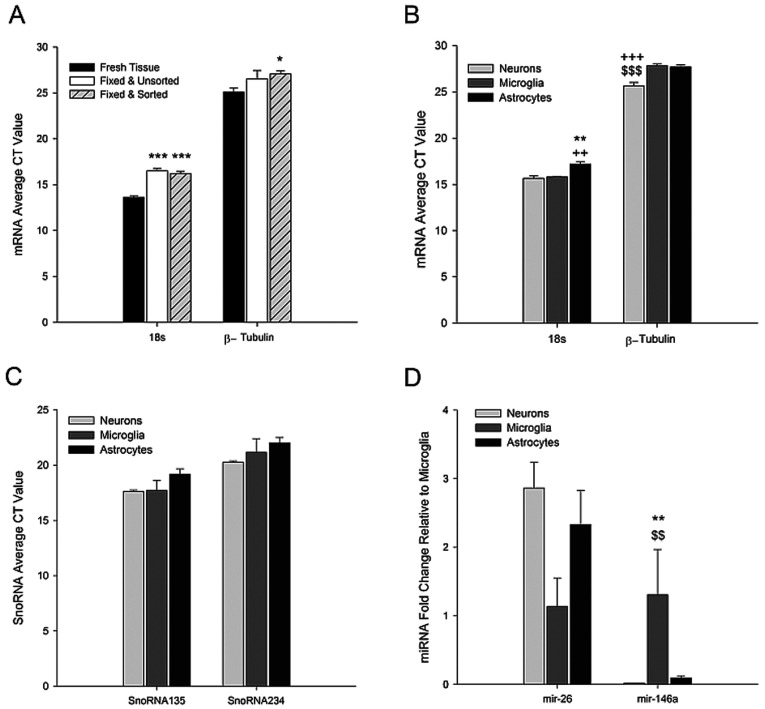
Retrieved RNAs from sorted cells are suitable for analysis by qRT-PCR RNA was harvested and qRT-PCR was used to analyze the levels of two commonly used housekeeping genes: 18s rRNA and β-tubulin mRNA. Comparisons were made between (**A**) fresh tissue, fixed/unsorted cells, and fixed/sorted cells (*n*=4–8) or (**B**) sorted neurons (NeuN^+^), microglia (CD11b^+^) and astrocytes (GFAP^+^) (*n*=8). (**C**) The expression of two commonly used housekeeping small non-coding RNAs (SnoRNA-135 and -234) and (**D**) two miRNAs miR-26 and miR-146a in neurons (NeuN^+^), microglia (CD11b^+^), and astrocytes (GLT1^+^) were also evaluated (*n*=4). Symbols indicate significant differences between samples as assessed by a one-way ANOVA.*versus fresh tissue (**A**) or neurons (**B**–**D**);+versus microglia; and $versus astrocytes. 1 symbol *P*<0.05; 2 symbols *P*<0.01; 3 symbols *P*<0.001.

We next sought to determine if we could isolate RNA from the mZBF-fixed and sorted cells. Table 2 indicates the average RNA concentrations obtained from 50 000 cells, and the purity of RNA obtained from each cell population based on the 260/280 ratios. To assess whether RNAs isolated from mZBF-fixed and sorted CNS cells was of suitable quality for downstream analyses, we utilized qRT-PCR to compare the levels of two commonly used ‘control genes’, the ribosomal RNA18s, and the messenger RNA, β-tubulin (*n*=8). We examined this in live cells (unfixed), and cells that were either fixed and unsorted, or fixed and sorted, to test the influence of both the fixation and sort processes. We found that the C_T_ values for both 18s and β-tubulin with zinc fixation were higher when compared to C_T_ values in live, unfixed cells (Figure 3A), indicating that RNA quality is somewhat reduced by the fixation process, or that there are factor(s) acting as PCR inhibitors. Interestingly, 18s RNA appears to be more negatively affected by the fixation process than β-tubulin, suggesting a potential RNA size effect. When compared to live cells, the C_T_ values for 18s were increased by ~3 cycles in both the fixed/unsorted (*P*<0.001) and fixed/sorted (*P*<0.001) samples, whereas β-tubulin was increased by ~1 cycle in the fixed/unsorted samples (*P* = 0.260) and by 2 cycles in the fixed/sorted samples (*P* = 0.024). This suggests that larger RNAs may be more susceptible to degradation and/or that they are more difficult to isolate than smaller RNAs. Although there is indication that the fixation process causes some RNA degradation/PCR inhibition, mRNA levels are readily detectable, and the sorting process does not appear to further compromise downstream analyses. We next compared the levels of 18s and β-tubulin among microglia, astrocytes and neurons (Figure 3B). We found that 18s levels were significantly lower in astrocytes (GFAP^+^) compared to levels in microglia (CD11b^+^; *P*<0.002) and neurons (NeuN^+^; *P* = 0.001). In addition, the expression of β-tubulin was significantly higher in neurons compared to microglia (*P*<0.001) and astrocytes (*P* = 0.001).

Since we were effective in retrieving both rRNA and mRNA from fixed/sorted cells, we next evaluated the compatibility of zinc fixation with the recovery of small RNAs in four independent samples derived from four separate animals. We screened two small non-coding nuclear RNAs that are often used as loading controls for miRNA qRT-PCR studies, snoRNA135 and snoRNA234. We successfully detected both snoRNAs (Figure 3C), and found no cell-type-specific differences in the levels of either (*P* = 0.278 and 0.463, respectively). Finally, we screened two miRNAs: miR-26 highly expressed in the CNS (Smirnova et al., 2005), and miR-146a, an important regulator of innate immune responses in microglia (Rom et al., 2010; Saba et al., 2012; Ponomarev et al., 2013). Although they did not reach statistical significance, we found that compared to expression levels in microglia, miR-26 tended to be 2–3-fold more highly expressed in neurons (*P* = 0.051) and astrocytes (*P* = 0.080). miR-26 expression levels were comparable in astrocytes and neurons (*P* = 0.696). miR-146a was highly expressed in microglia, but as expected, it was nearly undetectable in both astrocytes (*P* = 0.006) and neurons (*P* = 0.001).

It is unclear at this time if the differences in C_T_ values among cell types for 18s and β-tubulin are the result of cell-type-specific differences in expression of these RNAs, or if there are inherent differences in the quality of RNA harvested from different cell populations. We suspect the latter because similar differences in expression patterns were observed with two other housekeeping RNAs (snoRNA135 and snoRNA234), although they were not statistically significant. snoRNA135 was similar in pattern to 18s, whereas snoRNA234 was similar to β-tubulin. In addition, the quantity of RNA obtained from the same number of cells based on absorbance at 260 nm appeared to be 3–4 times higher for astrocytes compared to neurons and microglia (Table 2). To account for these differences in subsequent gene expression analyses, we averaged the 18s and β-tubulin C_T_ values for ∆C_T_ calculations.

### Cell-specific gene expression confirms purity of fixed, stained and sorted cells

**Figure 4:**
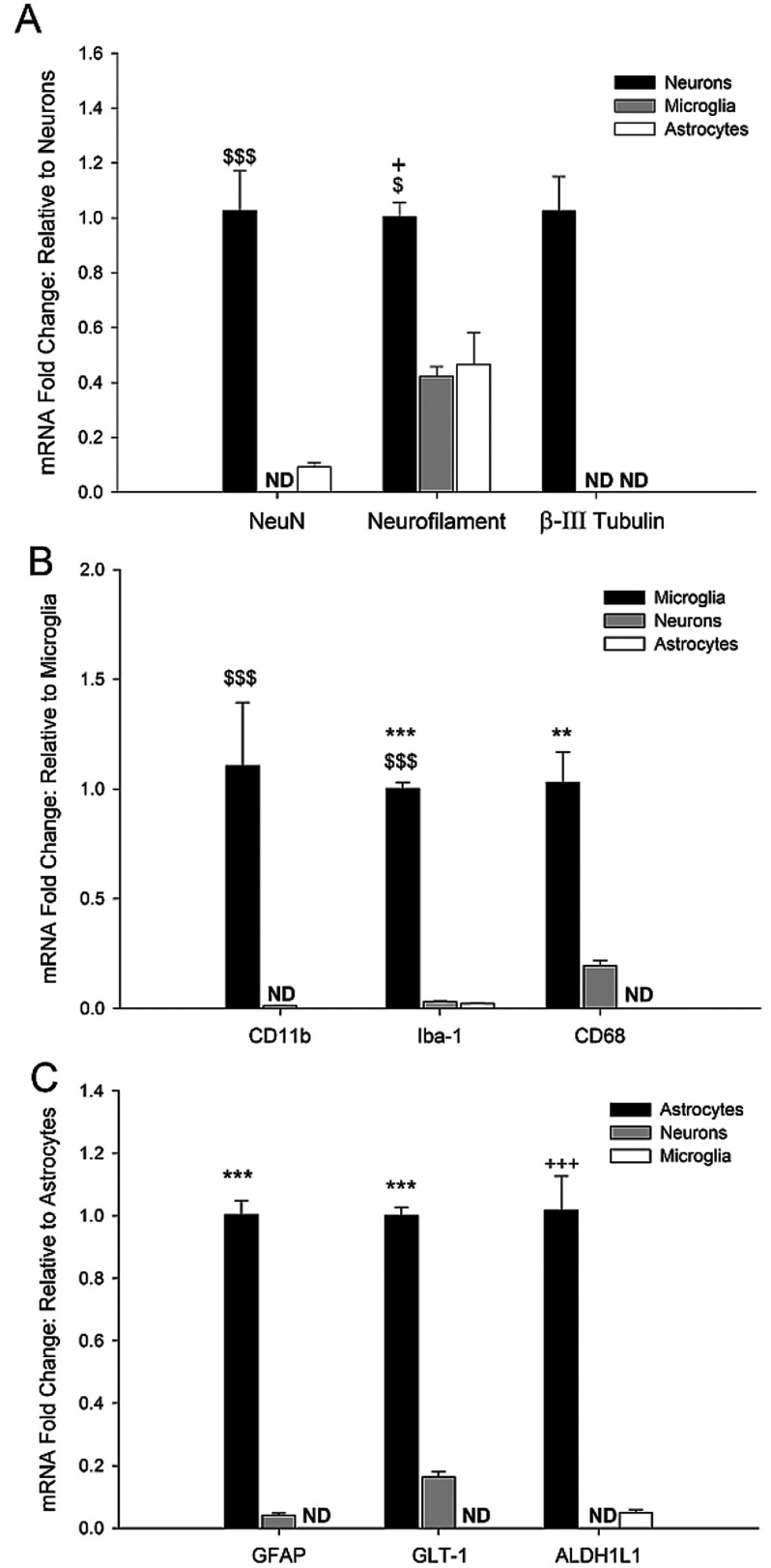
qRT-PCR of cell-specific genes confirms sorted CNS cell purity Total RNA harvested from fixed/sorted cells was utilized for qRT-PCR of cell-type specific genes to confirm the purity of the sort. (**A**) The neuron-specific genes NeuN, neurofilament, and β(III)- tubulin), (**B**) the microglia-specific genes CD11b, Iba-1 and CD68, and (**C**) the astrocyte-specific genes GFAP, GLT-1 and ALDH1L1 were evaluated in all three cell populations (*n*=4–8). Symbols indicate significant differences between samples as assessed by a one-way ANOVA.*versus neurons;+versus microglia; and $versus astrocytes. 1 symbol *P*<0.05; 2 symbols *P*<0.01; 3 symbols *P*<0.001.

Using qRT-PCR to analyze the expression of known cell-type-specific genes, we assessed the purity of sorted neuron, microglia and astrocyte cell populations from four independent cells sorts. We found that the neuron-specific genes NeuN, β-III tubulin and neurofilament were highly expressed in NeuN^+^ cells, and that NeuN and β-III tubulin were lowly expressed or undetectable in microglia and astrocytes (Figure 4A). Surprisingly, neurofilament was somewhat detectable in both microglia and astrocytes. The microglia-specific genes CD11b, Iba-1 and CD68 were highly expressed in microglial (CD11b^+^) cells, and lowly expressed or undetectable in astrocyte (GFAP^+^) and neuron (NeuN^+^) populations (Figure 4B). Likewise, the astrocyte-specific genes GFAP, GLT-1 and ALDH1L1 were highly expressed in GFAP^+^ cells (Figure 4C) and lowly expressed in microglia and neurons.

### Cell-type specific expression of Jumonji histone demethylases

**Figure 5:**
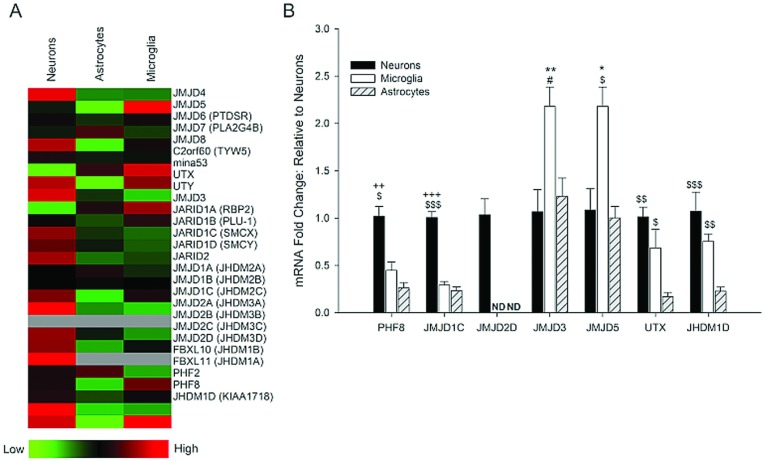
Jumonji histone demethylase gene expression is cell-type specific in the CNS (**A**) Total RNA was harvested from sorted cell populations, a portion of the cDNA was pooled, and screened in a custom-made qRT-PCR array consisting of 26 Jumonji histone demethylases. (**B**) Genes with a greater than 3-fold difference between cell types were confirmed by qRT-PCR in four independent samples. Symbols indicate significant differences between samples as assessed by a one-way ANOVA.*versus neurons;+versus microglia; $versus astrocytes; and # *P* = 0.060 versus astrocytes. 1 symbol *P*<0.05; 2 symbols *P*<0.01; 3 symbols *P*<0.001.

We used a custom qPCR array to analyze the expression of 26 JmjC-domain containing histone demethylases (Jumonji demethylases) in sorted microglia, neurons and astrocytes. For initial screening purposes, we pooled equal amounts of cDNA from four independent animal samples into a single sample (run in duplicate) to obtain the average expression of each Jumonji gene in all cell types. We found that 12 of the 26 Jumonji mRNAs were differentially expressed among cell types by at least 2–3-fold, and seven had greater than a 3-fold change relative to the other cell types (Figure 5A). We confirmed these results in samples from four independent cell sorts, obtained from four different animals, using qRT-PCR to profile the Jumonji mRNAs identified by the array as being the most differentially expressed in all three cell types: PHF8, JMJD2D, JMJD3, JMJD5, UTX, JHDM1D and JMJD1C (Figure 5B). We found the expression of PHF8, JMJD1C and JMJD2D to be highly specific to neurons. In addition, we found that JMJD3 and JMJD5 were primarily expressed in microglia (2.7±0.60 and 2.2±0.2-fold higher relative to neurons, respectively). Finally, UTX and JHDM1D mRNA levels were very low in astrocytes (0.17±0.04 and 0.23±0.04-fold relative to that in NeuN^+^ cells, respectively).

### Identification of histone-tail post-translational modifications by FCM

**Figure 6:**
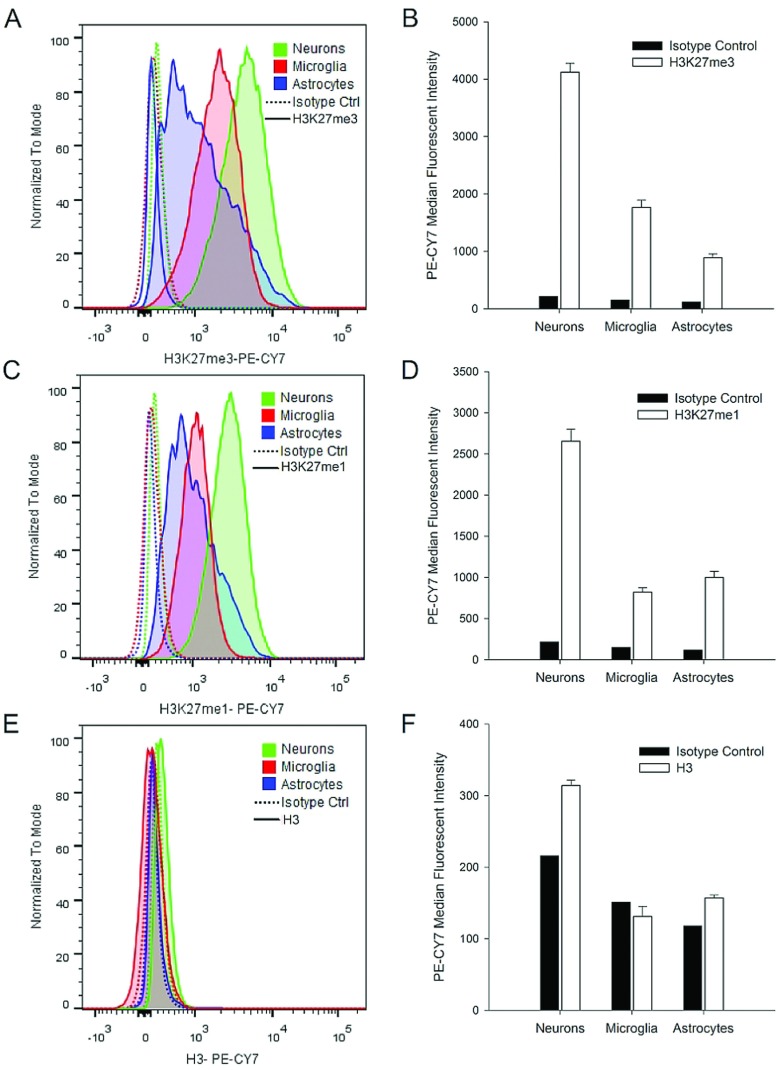
Post-translational histone modifications can be analyzed by FCM in zinc-fixed cells mZBF-fixed cell suspensions were stained with antibodies against NeuN, CD11b, and GFAP to identify neurons, microglia and astrocytes respectively, in the presence of antibodies against the histone mark (**A**,**B**) H3K27me1, (**C**,**D**) H3K27me3, or (**E**,**F**) pan-H3. Singlet and DAPI^+^ gated populations were subsequently gated by cell-type, and changes in the median fluorescent intensity (MFI) of the respective histone marks were assessed. Shifts in the MFI with primary antibody relative to signal with IgG isotype control antibody are shown in the histograms, and averaged data (*n*=4 independent samples) are shown in the bar graphs.

To determine if investigations of the functional role of Jumonji histone demethylases in sorted CNS cells could be performed using this method, we next assessed whether histone-tail post-translational modifications could be visualized by FCM in mZBF-fixed microglia, neurons and astrocytes. We co-stained cells with antibodies against H3K27me1 (Figures 6A and 6B) and H3K27me3 (Figures 6C and 6D), together with cell-specific markers. As expected, we detected both H3K27me3 and H3K27me1 in all cell types. However, neurons and astrocytes appeared to have increased and more heterogeneous expression of H3K27me1 and H3K27me3 than microglia as evidenced by broader peaks on the histogram. We also used an antibody against the H3 histone protein that recognizes an epitope on the C-terminus of the core histone not readily accessible in its native conformation. With this antibody, we observed only a slight shift in the mean fluorescent intensity (Figures 6E and 6F) although pan H3 immunostaining in microglia, in contrast to the methylated immunostaining at H3K27, appeared to be greater than in neurons and astrocytes. These results indicate that native histone structure is maintained in the mZBF-fixed cells, that histone tail post-translational modifications can be visualized by FCM in this fixative, and that levels of methylated histones differ among CNS cell types.

## DISCUSSION

To our knowledge, this is the first study to successfully identify and isolate distinct CNS neuron and glial cell populations simultaneously from the adult, non-transgenic, rodent brain based on a combination of intra- and extra-cellular markers by FCM. The mZBF used here preserves cell surface, intracellular, and nuclear protein structure, and we have used it to perform analyses of cell cycle, histone tail modifications, and other proteins prior to cell sorting, maximizing the information obtainable from a single CNS tissue sample. We show that the quality of ribosomal, messenger and miRNAs retrieved from the sorted cells is adequate for subsequent downstream qRT-PCR analyses, providing a means to study epigenetic regulation of gene expression in the specific CNS populations from the same sample. This important technical advance represents a particularly significant step forward for gene expression studies in non-neuronal cell types for which intracellular epitopes are the best characterized, and/or for which antibodies against extracellular epitopes of cell-type-specific surface markers are not widely available. An additional benefit of this method is the ability to store samples for many weeks for later analyses.

Cellular fixation and permeabilization are necessary for antibody-mediated detection of intracellular antigens by FCM. Recovery of RNAs from standard formaldehyde- or paraformaldehyde-fixed cells is poor (Esser et al., 1995; Diez et al., 1999) especially when cell numbers are limiting, and alcohol fixation is not ideal for extracellular marker-based cell identification [results not shown and (Shapiro, 1988; Perez et al., 2001)]. Several recent studies have successfully used an alcohol-based fixation method for recovery of RNA from intracellularly identified neuron populations (Guez-Barber et al., 2011b; Fanous et al., 2013; Liu et al., 2014). However, because we wished to use a combination of intracellular and cell surface markers for cell identification, we applied the modified zinc-based fixation method to isolate RNAs from fixed and sorted neurons and glia. Consistent with previous reports (Wester et al., 2003; Lykidis et al., 2007; Jensen et al., 2010), DNA could also be successfully isolated from zinc-fixed cells (results not shown), increasing the utility of this technique for the study of epigenetic DNA modifications. Many fields are plagued by the need/desire to fix samples prior to flow cytometric analysis and/or cell sorting, and more information can be obtained from fixed cells (because intracellular proteins can be quantified). Fixation often adds a level of safety for more biohazardous samples since significant biosafety concerns are present when working with human, pathogen-infected or virally transduced cells, particularly in FCM facilities where the potential for aerosolization is high (Schmid et al., 2003). Although not tested here, this fixation method may also be a useful alternative to live sorting when nucleic acid recovery is desired from biohazardous samples.

We undertook the present study to begin to investigate the epigenetic control of gene expression in microglia and neurons as it relates to cell–cell communication in pathology. Although live microglia can easily be identified and sorted based on cell surface marker identification (e.g. CD11b) (Nikodemova and Watters, 2012), the same cannot be done with live neurons, as the best-characterized markers are intracellular. Adult astrocyte identification also suffers from the same issues as neurons with regard to intracellular markers and/or antibodies only being available toward intracellular epitopes of cell surface markers (e.g. GLT-1). Indeed, the dissociation procedure reported here is not optimized for astrocytes as the mechanical disruption decreases astrocyte viability, reducing their recovery. However, the mechanical method was optimal for the retrieval of neurons and microglia, which are the major cell types of interest to us. In the course of optimizing this technique for our purposes, we found that enzymatic dissociation with papain (Nikodemova and Watters, 2012) permitted much better astrocyte recovery, although it greatly decreased neuron retrieval (results not shown). Efficiency of microglial recovery with both methods was not different. The overlap observed between the GFAP^+^ and NeuN^+^ cells (<1%; Figure 2C) is probably a product of the mechanical dissociation process. For example, incomplete dissociation may result in astrocyte debris sticking to neuronal cells. Alternatively, GFAP/NeuN double positive cells could represent a population of neural progenitor cells expressing both NeuN and GFAP markers (Sanchez-Ramos et al., 2000). Results from the ImageStream analyses supported both notions: whereas there were true NeuN^+^/GFAP^+^ double positive cells (likely precursor cells), the most were double positive because of cell debris stuck to an intact cell (results not shown). In addition, there was a large proportion of cells (~39%) that were triple negative for the three cell identification markers used here. Although additional studies are needed to specifically identify the members of this cell population, we speculate that these are likely to be astrocyte/glial cells and endothelial cells that were not identified by the primary markers used here. For example, whereas GFAP is the most commonly used marker for astrocytes, it is only expressed by a fraction of astrocytes, the intensity and percentage of which varies throughout the brain (Kimelberg, 2004). Thus, while the present dissociation method is not ideal for GFAP^+^ astrocyte recovery, more efficient and universal astrocyte identification may require simultaneous staining with multiple astrocyte markers (e.g. GFAP, GLT-1, ALDH1L1), regardless of dissociation method.

Because histone demethylation is commonly associated with the activation/repression of gene transcription (Takeuchi et al., 2006; Kooistra and Helin, 2012), and many microglial inflammatory activities require new gene transcription of cytokines and other mediators of inflammation (Blackwell and Christman, 1997; Doyle and O’Neill, 2006), we were particularly interested in which histone demethylase enzymes were expressed in microglia and neurons. In this study, we focused on the Jumonji histone demethylase gene family that contains 30 members based on sequence homology (Kooistra and Helin, 2012). The Jumonji demethylases catalyze a dioxygenase reaction to remove mono-, di- and tri-methyl groups from lysine and arginine residues, primarily on histone H3, H4 and H1 substrates. This reaction is dependent on Fe (II) and α-ketoglutarate (Kooistra and Helin, 2012). There is very little known about the role of Jumonji histone demethylases in the regulation of CNS function in general, and nothing is known about their cell-specific expression in the adult CNS. Given the significant differences in gene expression profiles in microglia, neurons and astrocytes, we hypothesized that there would be cell-type-specific differences in the basal expression of the Jumonji demethylase genes.

We found that seven Jumonji family genes were most differentially expressed between neurons, microglia and astrocytes. PHF8, JMJD1C and JMJD2D were highly neuron-specific as they were undetectable or very lowly expressed in microglia and astrocytes. Neuronal expression of PHF8 and JMJD1C is consistent with previous reports (Wolf et al., 2007; Qiu et al., 2010). PHF8 is important for proper neural development (Qiu et al., 2010), and its mutation is associated with Fragile X syndrome, believed in part to be responsible for the associated cognitive deficits (Laumonnier et al., 2005; Kleine-Kohlbrecher et al., 2010). JMJD1C protein levels are high in androgen-responsive neurons where it is proposed to be involved in the co-activation of the androgen receptor (Wolf et al., 2007). Although mutations in the JMJD1C gene are linked to the development of autism (Castermans et al., 2007; Shulha et al., 2012), this relationship is not fully understood as the histone substrate for this enzyme has not yet been identified (Kooistra and Helin, 2012). Very little is known about the role of JMJD2D in the CNS. However, similar to JMJD1C, JMJD2D acts as an androgen receptor co-activator in the prostate (Shin and Janknecht, 2007). Perhaps in the CNS, JMJD2D plays a similar role to JMJD1C. We found that JMJD3 and JMJD5 were more highly expressed in microglia than in astrocytes or neurons. JMJD3 is an important regulator of both inflammatory (De Santa et al., 2009; Das et al., 2010; Luo et al., 2011) and anti-inflammatory activities in macrophages (Satoh et al., 2010), and JMJD5 expression in osteoclasts is an important regulator of osteoclastogenesis (Youn et al., 2012). There is now one report of a Jumonji gene family member (JMJD3) in microglia (Tang et al., 2014). JMJD3 was shown to be critical for switching between inflammatory and anti-inflammatory/reparative phenotypes in immortalized microglia, consistent with reported effects in macrophages. Finally, we found UTX and JHDM1D to be more lowly expressed in astrocytes, compared to neurons and microglia. UTX is an X-chromosome-linked gene and is more highly expressed in the female brain (Xu et al., 2008), but nothing is known about its expression in astrocytes. Only male rats were included in our study; thus, it is possible that UTX levels may be higher in astrocytes isolated from female rats. Interestingly, a point mutation in the UTX gene results in Kabuki syndrome (Lederer et al., 2012; Miyake et al., 2013) characterized by developmental delay, cognitive disabilities and craniofacial abnormalities. Because these morbidities are also associated with aberrant astrocyte activity (e.g. Alexander disease, Fragile X disease) (Molofsky et al., 2012), impaired or overactive UTX activity in astrocytes specifically, may play an important physiological role in neuropsychiatric disease. Lastly, little information is available on the role of JHDM1D in the brain. It is known to regulate neural differentiation (Huang et al., 2010b) and neural fate specification (Huang et al., 2010a) during embryogenesis. We find its expression in the adult CNS to be lowest in astrocytes and highest in neurons, consistent with the idea that the JHDM1D gene may be down-regulated in astrocytes after progenitors have committed to the glial phenotype. Whether differences in basal Jumonji demethylase gene expression contribute to cell-type-specific regulation of gene expression in the adult CNS is not yet known, but the present data are consistent with this possibility.

There are apparent differences in the magnitude of H3K27me1 and H3K27me3 among CNS cell types, with neurons having the highest expression of both marks. While the significance of this is not yet clear, differences in H3K27 methylation at the specific gene promoters may contribute to fundamental, cell-specific differences in gene expression profiles among these cell types. Additional studies involving chromatin immunoprecipitations are necessary to test this hypothesis further. In addition to histone modifications, miRNAs are another important aspect of epigenetic gene regulation. In this study, we evaluated levels of miR-146a and miR-26 in sorted microglia, astrocytes and neurons to determine if the quality of recovered miRNAs from cells processed in this way was adequate for subsequent investigation. While we could easily amplify miRNAs, we found cell-type specificity of the two miRNAs we evaluated. We found that miR-146a was highly expressed in microglia (but was very low in neurons and astrocytes), consistent with its expression primarily in myeloid lineage cells (O’Connell et al., 2011; Ponomarev et al., 2013). Because miR-26 is reported to be preferentially expressed in astrocytes (Smirnova et al., 2005), we were surprised to find it equally detectable in astrocytes and neurons. However, the astrocyte-specific expression of miR-26 was determined in differentiated murine stem cells *in vitro* (Smirnova et al., 2005). Our samples are rat, and there are significant species differences in miRNAs. Alternatively, differences may be related to cell age. Our studies were performed on adult cells isolated from the whole brain; stem cells differentiated *in vitro* may not accurately reflect identical gene expression levels and patterns of 3-month-old adult cells *in vivo*. Furthermore, gene expression in neurons and astrocytes *in vivo* are highly influenced by the CNS microenvironment and cannot be identically recapitulated *in vitro* during stem cell differentiation. Together, these results reinforce the need to study miRNAs in specific cell types *in vivo*.

In conclusion, this technique adapts known FCM and cell fixation methods to the field of neuroscience, advancing our ability to study epigenetics and gene regulation simultaneously in glia and neurons. To lay the foundation for future studies of histone demethylation-regulated gene expression, we utilized this procedure to investigate the profile of Jumonji gene family histone demethylase expression in neurons, microglia and astrocytes from the healthy CNS. Our findings are consistent with the limited available literature. More importantly, the results provide new information about cell-specific Jumonji gene expression in the adult CNS, underscoring the importance of understanding cell-specific regulation of epigenetic modifiers in the adult, non-transgenic brain.
